# A portable battery-powered flow injection monitor for the in situ analysis of nitrate in natural waters

**DOI:** 10.1155/S1463924693000215

**Published:** 1993

**Authors:** N. J. Blundell, A. Hopkins, P. J. Worsfold, H. Casey

**Affiliations:** 1 Department of Environmental Sciences University of Plymouth Plymouth PL4 8AA UK; 2 Institute qf Freshwater Ecology River Laboratory East Stoke Wareham, Dorset BH20 6BB UK

## Abstract

The design and performance of a portable, automated flow injection (FI)-based photometric monitor are described. The system is controlled by an in-house microcomputer system that enables the monitor (including a solid state detector) to operate from a 12 V battery supply. The monitor uses the cadmium reduction/diazotization method to analyse for nitrate with a linear range of 0 to 12 mg l^-1^ and a limit of detection of 0.05 mg l^-1^ (NO_3_-N). The hardware and software design, monitor performance and results obtained during unattended operation are presented.


Journal of Automatic Chemistry, Vol. 15, No. 5 (September-October 1993), pp. 159-166

### A portable battery-powered flow injection monitor for the *in situ* analysis of nitrate in natural waters

**N. J. Blundell, A. Hopkins, P. J. Worsfold**

*Department of Environmental Sciences, University of Plymouth, Plymouth PL4 8AA, UK*

**and H. Casey**

*Institute of Freshwater Ecology, River Laboratory, East Stoke, Wareham, Dorset BH20 6BB, UK*

*The design and performance of a portable, automated flow injection (FI)-based photometric monitor are described. The system is controlled by an in-house microcomputer system that enables the monitor (including a solid state detector) to operate from a 12 V battery supply. The monitor uses the cadmium reduction/diazotization method to analyse for nitrate with a linear range of 0 to 12 mg $l^{-1}$ and a limit of detection of 0·05 mg $l^{-1}$ ($NO_3$-N). The hardware and software design, monitor performance and results obtained during unattended operation are presented.*

### Introduction

Physical water quality parameters, such as pH, temperature, flow rate and conductivity, can be routinely determined *in situ* using the appropriate sensor and a data logging device. A limited number of chemical parameters (for example ammonia and nitrate) can also be measured, using ion selective electrodes, but such devices can often suffer from electrode fouling and require frequent recalibration to maintain accuracy.

The conventional method of measuring chemical parameters, however, involves batch sampling in the field with subsequent laboratory analysis. Potentially, this procedure can introduce errors into the process due to contamination and degradation of the sample between taking the sample and analysis [1] and becomes very time consuming and labour intensive, particularly when large numbers of samples are involved.

There is a growing need to conduct on-line chemical analyses in the field to provide a pseudo-continuous profile of water quality parameters. This is particularly important for those species (for example, nitrate) which are covered by EC legislation [2], because the conventional (manual) monitoring scheme may not detect short-term changes (for instance storm events, or point discharges) between sampling events.

Flow injection (FI) is ideally suited to automating standard laboratory methods and has been successfully applied to process control [3] and environmental monitoring [4, 5]. In the latter context field monitors have been developed for such parameters as nitrate [6], phosphate [7], ammonia [8] and aluminium [9] using solid state photometric detection [10]. These designs were based on commercial mains-powered microcomputer systems, and are thus unsuitable for use in a portable (i.e. battery-powered) monitor.

Recent developments in microchip technology have led to the production of specialized microcontroller devices for control and automation applications. Such devices have many of the necessary I/O functions built onto the chip and consequently many of the peripheral devices associated with microcomputer-based systems are no longer required, allowing miniaturization of system hardware. The advantages of microcontroller-based systems are particularly clear for battery-powered equipment which can be constructed from a relatively small number of low power CMOS components. Thus, microcontroller-based systems have been used in applications such as data logging [11], laboratory automation [12] and *in situ* monitoring [13].

This paper describes the construction of a battery-powered automated FI monitor, comprising an in-house designed microcomputer and solid state detection system. The microchip used is a member of the industry standard Intel 8051 series of microcontrollers which are widely employed in industrial control applications, such as anti-skid braking systems and engine management systems. The computer system described has been designed to automate all of the functions required for field-based operation, i.e. control of peristaltic pumps, injection valve and switching valve and data acquisition, processing and logging. An FI manifold for nitrate has been installed in the monitor and data are reported from laboratory stability trials and nitrate fluctuations in tapwater.

### Experimental

### *Reagents*

All solutions were prepared using Milli-Q water (Millipore). Reagents were AnalaR (Merck), except N-(1-naphthyl)ethylenediamine dihydrochloride (Sigma) and 100 mesh cadmium powder (Johnson Matthey). A 1000 mg $l^{-1}$ $NO_3$-N stock solution was prepared by dissolving 7·220 g of potassium nitrate (dried for 2 h at 375 K) in 1 l of water. Working nitrate standard solutions were prepared by serial dilution of this stock solution. The ammonium chloride carrier stream was prepared by dissolving 10 g ammonium chloride in 1 l of water. The sulphanilamide reagent was prepared by dissolving 25 g of the compound in 1 l of 10% (v/v) orthophosphoric acid. The N-(1-naphthyl)ethylenediamine dihydrochloride (N1NED) reagent was prepared by dissolving 0·5 g of the solid in 1 l of 10% (v/v) orthophosphoric acid. A working

0142-0453/93 $10.00 © 1993 Taylor & Francis Ltd.

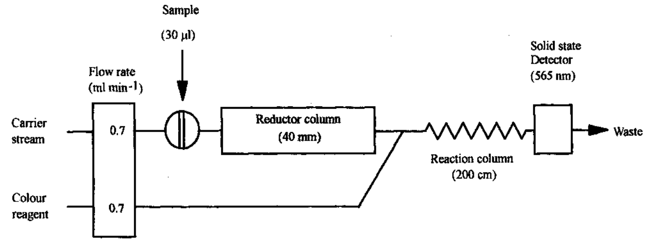

*Figure 1. Optimized manifold for the determination of nitrate in freshwaters [6].*

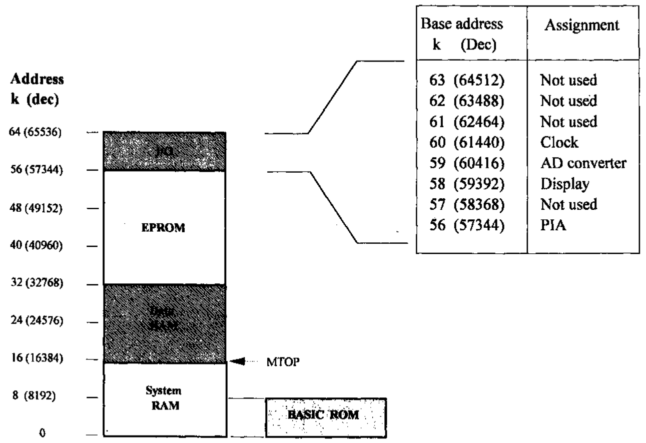

*Figure 2. Memory map of the 80C32 microcontroller.*

colour reagent was prepared by mixing equal volumes of the N1NED and sulphanilamide reagents. The reagents were stored in plastic-ware containers, except the mixed colour reagent which was stored in a brown glass bottle.

Cadmium reductor columns were prepared by adding 5 g of cadmium power (100 mesh) to 50 ml of a copper (II) sulphate solution (0·25 g) and stirring for 2 minutes. The resulting copperized cadmium was washed with 2 M hydrochloric acid (2 × 50 ml) and ammonium chloride solution (10 g $l^{-1}$). The slurry was then packed into glass tubes (50 mm × 3 mm i.d.) and plugged with glass wool. The columns were connected to the PTFE tubing by 20 mm lengths of orange/orange pump tubing (Anachem).

A concentrated diazo dye solution was prepared by mixing 5 ml of a 50 mg $l^{-1}$ $NO_2$-N solution (itself prepared by serial dilution of the 1000 mg $l^{-1}$ stock solution) and 20 ml each of the mixed colour reagent and ammonium chloride solutions. The absorbance standards used for the calibration of the solid state detector were prepared by serial dilution of this intensely coloured solution. Absorbance data for this comparison were measured at 565 nm using an LKB Ultrospec II UV/visible spectrophotometer fitted with an 18 µl glass flow cell with a 10 mm path length (Hellma).

### *Chemical method and FI manifold*

The analytical procedure used for the chemical determination of nitrate is an adaptation of a standard water industry method [14]. It involves the reduction of nitrate to nitrite by a copperized cadmium mini-column followed by derivatization with sulphanilamide and coupling with N-(1-naphthyl)ethylenediamine dihydrochloride. The diazo product can be detected spectrophotometrically ($\varepsilon_{542} = 4{\cdot}6 \times 10^4$ $dm^3$ $mol^{-1}$ $cm^{-1}$).

The manifold design (see figure 1) is based on that reported previously [6] and is constructed from 0·8 mm i.d. PTFE tubing (Anachem) and in-house PTFE T-pieces.

### *Computer system*

The hardware and software were developed and debugged on a platform based around the 8-bit Intel 8052AH BASIC microcontroller [15]. Some of the features which make this device particularly attractive for developing intelligent instrumentation include:

(1) Programmable in BASIC.

(2) Built-in EPROM programming facility.

(3) Auto boot facility.

(4) Floating point arithmetic.

(5) Internal/external interrupts.

(6) Built-in clock.

The system has been designed with 32 k of battery backed RAM and 24 k EPROM space. The EPROM allows permanent storage of BASIC control and utility software and in addition, can store the baud rate, an auto boot flag and moveable system pointer (MTOP).

The boundary between the program/system and data sections of RAM is controlled by MTOP which allows a very flexible (software configurable) memory map. On initialization (power-up) the processor clears all memory locations up to the MTOP leaving locations above this suitable for storing logged data. Generally, once programs are running from EPROM, less than 4 k of RAM is required for the stack and variable storage. The system can therefore be configured with between 0 and 28 k non-volatile data storage space. For this application the MTOP is set at 16 384 (16 k).

Integrated into the CPU board design is a real time clock which is battery backed against external power failure and provides the system with access to the time, date, day of week, month and year. In addition, the clock has 50 bytes of RAM which are used by the control software to store the system configuration. The memory map of the computer system is illustrated in figure 2.

### *System I/O*

The microprocessor board is connected via a 40-way edge connector to a second Eurosized board which houses all of the system I/O components. Central to this second board is an 82C55 peripheral interface adapter (PIA) which is configured as three 8 bit TTL output ports. The first port (A) is connected to a network of eight relays which supply 12 V outputs to the rotary injection valve (Burkard Scientific) and the two-way solenoid switching valve (Biochem) and a 5 V supply to each of the pumps (Ismatec). The regulated 5 V supply drives each pump at a constant rate of 20 r.p.m. The relays are configured so that the pump direction can be reversed independently,

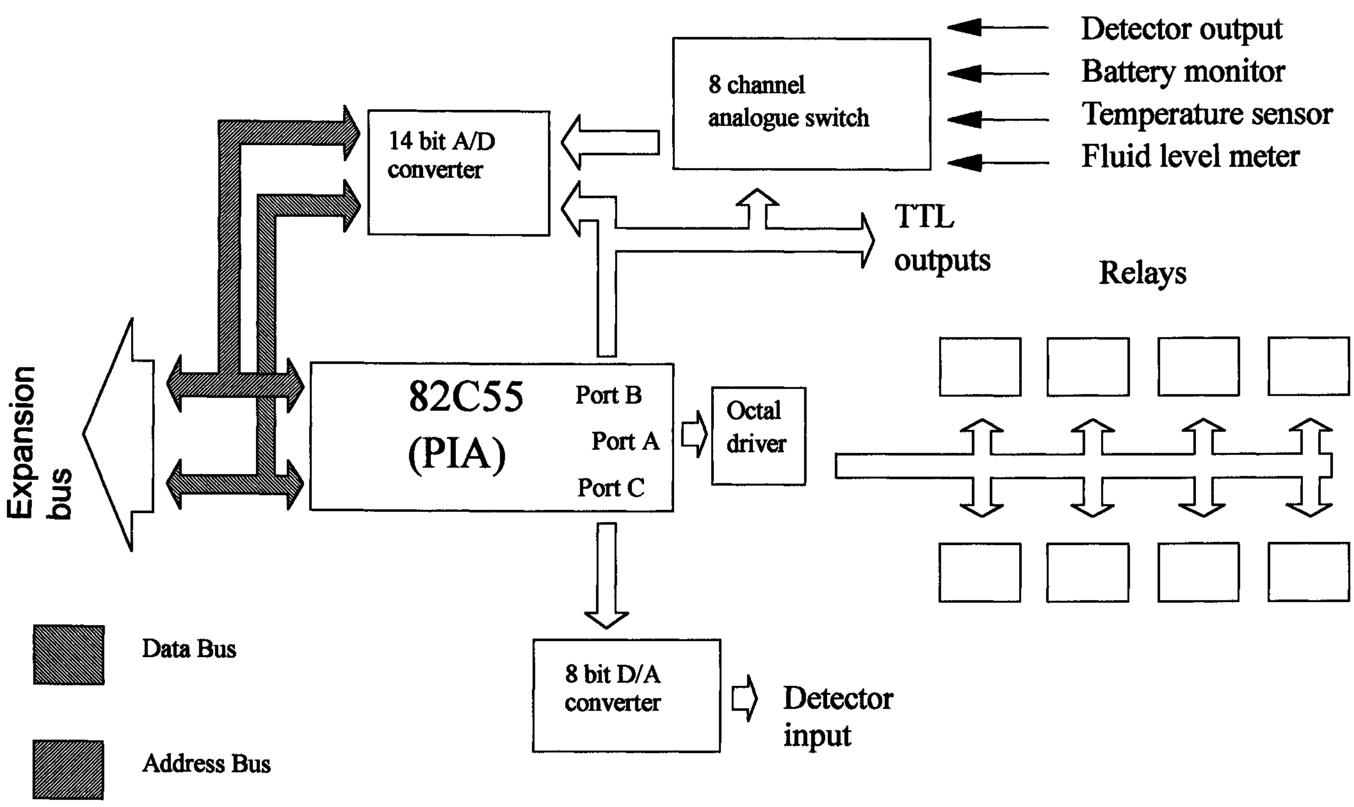

*Figure 3. Schematic representation of the I/O board.*

allowing each sample line to be back flushed if required. The second port (B) of the PIA supplies TTL triggers to the injection valve (to toggle between fill and inject positions), the 14 bit AD converter and the analogue switch. The final port (C) supplies the input to the 8 bit DA converter.

In addition to the detector output, the eight channel analogue switch enables the AD converter to monitor a series of sensors. These enable the computer to monitor the reagent levels and battery capacity and close the system down if either fall too low. A schematic representation of the I/O board is given in figure 3.

External communication is achieved by a three line RS232 connection to a PC. This can be either locally by direct connection or remotely using a pair of modems and a cellular phone link. The microcontroller communicates using an 8 bit data word, 1 stop bit and no parity and is capable of all commonly used baud rates up to 19 200. The serial link operates without hardware handshaking, so a baud rate of 9600 was selected as the most convenient rate without data loss.

An LCD screen (two lines of 20 characters) is connected to the system and displays simple messages describing the monitor status.

Once the system had been fully tested and the control software debugged and programmed into EPROM, the 8052 AH BASIC chip was replaced with a CMOS 80C32 microcontroller and the MCS BASIC v1.1 interpreter programmed into an external 8 k EPROM. Functionally, this configuration is identical to the initial set-up but the power consumption is reduced from about 400 to 40 mA.

*Solid state detector*

Solid state photometric detectors incorporate light emitting diodes (LEDs) as the light source and either photodiodes or phototransistors as the light detecting component. They offer several advantages over conventional spectrophotometers, most notably they are considerably smaller and consume less power.

The detector developed is a single channel device (consisting of a single LED and photodiode) which allows the power consumption to be reduced even further in comparison to a dual beam system. The photodiode (Radio Spares 308-067) has a spectral range of 200 to 1100 nm and includes an integral logarithmic amplifier. The voltage output of the device is linearly related to a function of the absorbance of the solution. A variety of LEDs are commercially available covering the majority of the visible and near infrared (NIR) regions of the spectrum (400–1100 nm) [10]. The most commonly encountered types include NIR ($\lambda_{max}$ = 950 nm), red ($\lambda_{max}$ = 635 nm), yellow ($\lambda_{max}$ = 583 nm), green ($\lambda_{max}$ = 565 nm) and blue ($\lambda_{max}$ = 470 nm) devices, which enable a range of analytes to be measured by choosing the appropriate LED [16]. The green LED used in this study is a 5 mm high intensity (200 mcd) type with an output wavelength close enough to the analyte absorption band ($\lambda_{max}$ = 562 nm) to allow detection without a significant loss in sensitivity.

The output from the photodiode is amplified by a precision operational amplifier (IC2), processed and passed through the analogue switch into the 14 bit AD converter. The AD converter includes an internal 3V reference giving the device a resolution of 0·2 mV. The

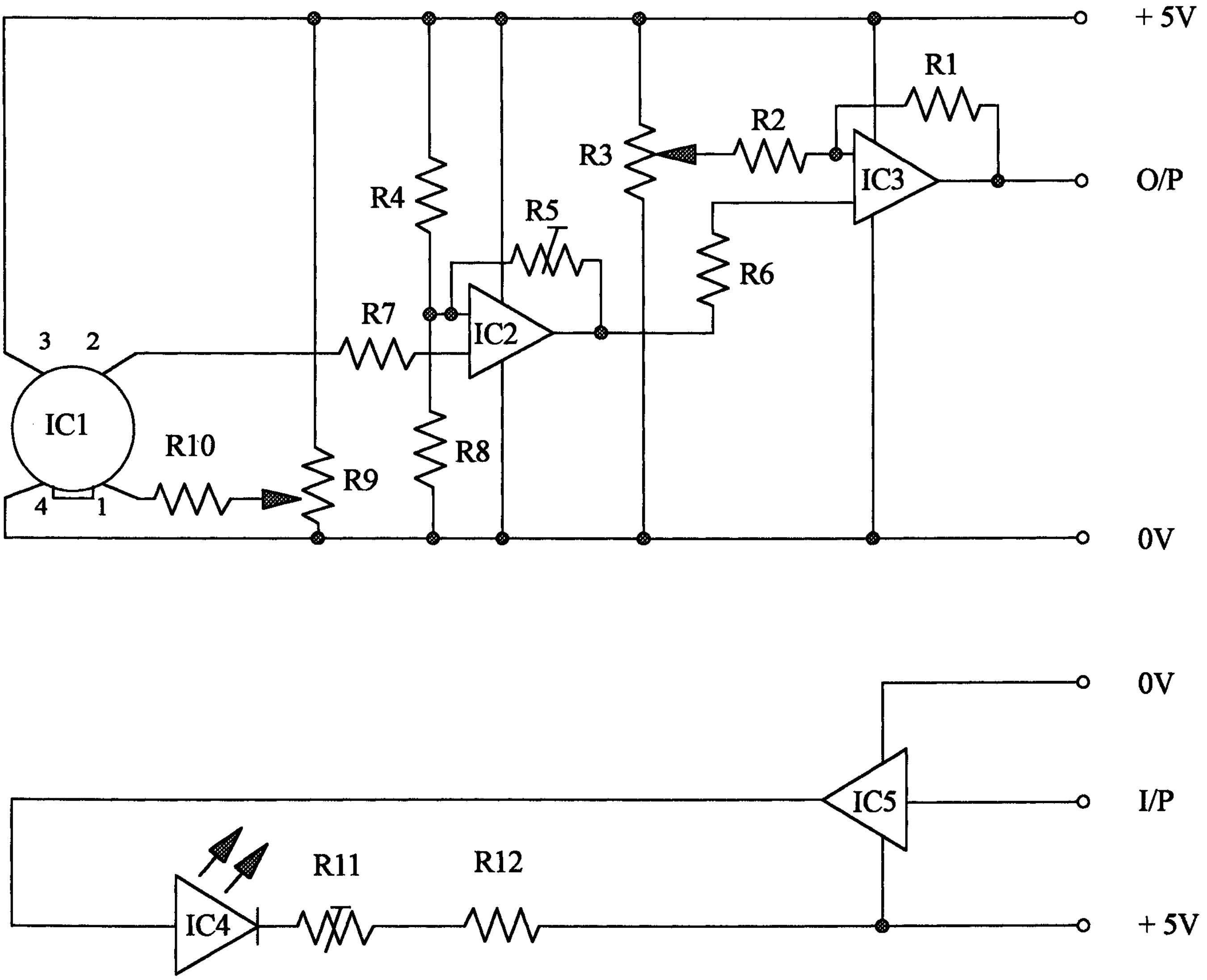

*Figure 4. Solid state detector circuit (R1 = 10 k, R2 = 10 k, R3 = 100 k, R4 = 27 k, R5 = 100 k, R6 = 4·7 k, R7 = 4·7 k, R8 = 27 k, R9 = 10 k, R10 = 10 k, R11 = 100, R12 = 68, IC1 = photo diode, IC2 = 911 op-amp, IC3 = 911 op-amp, IC4 = LED, IC5 = LN759 op-amp).*

detector circuit is integrated into the I/O board of the system and is shown in figure 4.

The detector includes an automatic baseline correction facility that enables the computer to adjust the detector baseline to within a software defined window at the beginning of each injection cycle. This is particularly important for a single channel design, which, unlike a dual channel detector, does not include internal compensation for instrumental drift during periods of inactivity.

*Flow cell*

The flow cell is of in-house design and construction and is a single channel derivative of that described previously [7]. The flow cell block, machined from aluminium (20 mm cube) consists of a length of PTFE tubing (2 mm i.d.) running orthogonal to the light path, which is held in position by 10 mm lengths of silicon tubing either side of the block. The detector is extremely sensitive to external light, so black heatshrink tubing is used to shield 10 cm lengths of the PTFE tubing on either side of the flow cell block. The flow cell block is securely mounted inside an aluminium box.

*Power supply*

The monitor is powered by 6 × 2 V (25 Ah) Cyclon cells (Radio Spares 591–629) connected in series. The batteries are sealed lead acid cells and were selected as they offered the best compromise between robustness and their storage capacity/size ratio.

*Control software*

The monitor is controlled by a BASIC program written in-house using the embedded Intel MCS BASIC interpreter (v1.1). The software is stored in EPROM and is configured to execute automatically on power-up. The monitor operation is configured by a series of system variables which define the injection cycle, for example,

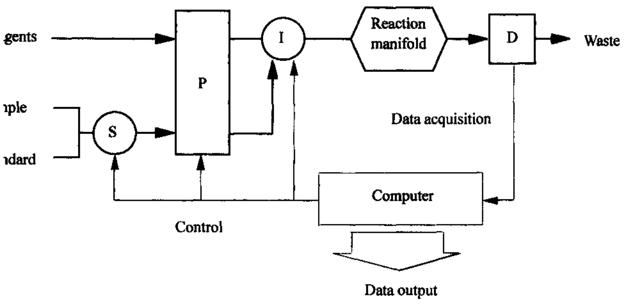

*'gure 5. Schematic of the automated FI monitor (S = Switching lve, P = Peristaltic pumps, I = Injection valve, D = Solid ıte detector).*

.e fill and delay times, the self-diagnostic routines and e data logging pointer. These data are stored in battery ıcked RAM and assigned to the relevant system ıriables on execution of the central control software. This :tends the flexibility of the system by allowing the system be reconfigured (for instance to alter delay times, AD tegrations), without needing to reprogram an EPROM.

### esults and discussion

### *fonitor operation*

ı addition to the standard laboratory requirements of :curacy and precision, long-term stability and some form ' automatic recalibration are desirable features for :ld-based instrumentation. The monitor described here flects these needs and is configured so that each ıalytical result is validated by the software and cali-ˆated against an on-board standard. A schematic presentation of the monitor design is shown in figure 5.

he analytical cycle involves duplicate injections of both ıe sample and on-board standard. Each set of duplicate jections is validated before the concentration of the .mple is calculated by a single point calibration. This ılibration method reduces the hardware and software ımplexity and power and reagent consumption in ımparison with a multipoint calibration. For an accurate :sult, however, the calibration must be linear over the ıtire concentration range and the intercept must pass ırough (or close to) the origin. It is therefore important ıat the blank signal is zero (or very close to zero), :herwise the divergence between the observed and redicted calibration slopes will introduce an increasingly rge error into the calculated value for the unknown as ıe actual concentrations of the sample and standard ıove apart. A flow diagram of the analytical cycle is ıown in figure 6.

### *'ata acquisition and processing*

he detector output is sampled at the maximum (software :stricted) rate of 15 Hz. The signal is averaged over a ıftware defined number of integrations, which ensures ıat the best compromise between reducing the noise of ıe detected signal and the rate of the reading required ı adequately cover the FI peak is achieved. After each

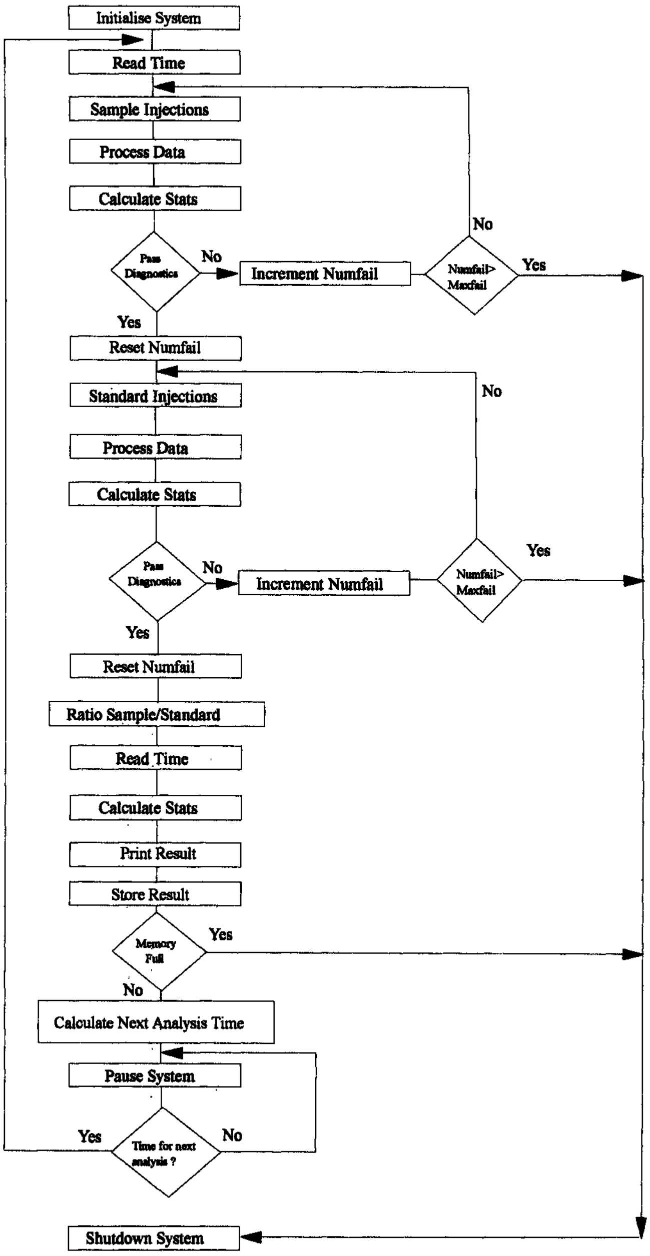

*Figure 6. Flow diagram of the control software.*

integrated value has been calculated it is stored as a segment in a 250 element array. Typically, for a 60 s injection window, each of the stored values represents the average of six conversions.

After data acquisition is complete the array containing the FI peak is digitally filtered using a moving median algorithm. Statistically, this filter is robust and non-parametric and is ideal for removing impulse noise from digital signals [17]. It is used widely in image processing [18] and has also been proposed as a method for correcting baseline drift in liquid chromatography [19]. The filter design preferentially removes sharper peaks and passes broader features; its discrimination between sharp and broad features is controlled by the filter window width, with peaks that span less than half of the window effectively removed from the output array. A seven segment window was found to be the optimum value for

*Table 1. Solid state detector calibration data.*

| | | | | | | | | | | | |
|---|---|---|---|---|---|---|---|---|---|---|---|
| Absorbance[a] | 0·000 | 0·018 | 0·044 | 0·106 | 0·156 | 0·341 | 0·448 | 0·655 | 0·950 | 1·328 | 2·440 |
| SSD response (counts) | 0 | 34 | 73 | 175 | 246 | 500 | 685 | 960 | 1415 | 1900 | 3600 |

[a] Measured at 565 nm.

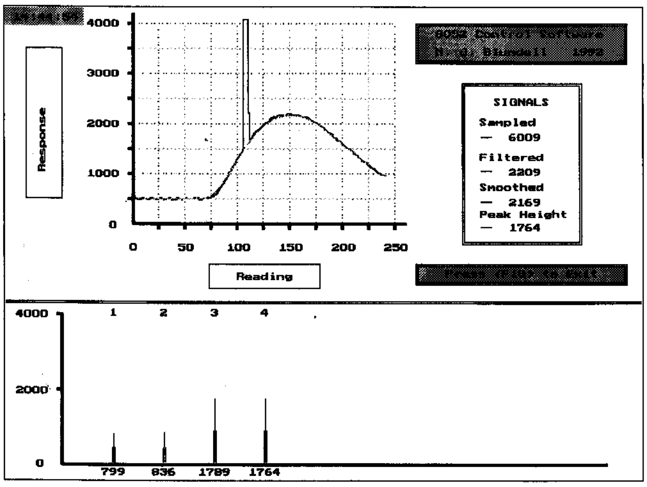

*Figure 7. Screen dump illustrating the effect of the data processing software on the detector signal.*

suppressing impulse noise caused by air bubbles in the FI stream. Larger filter ranks were found to degrade the sharper features of the FI peak and increase the processing time without any significant increase in the effectiveness of the filter operation.

Implementation of the algorithm involved sampling a moving window within the main data array. The data within the window are sorted into increasing numerical order and the median (middle) value identified. The central element is then reset to the median value and the window position incremented by one.

The second stage of data processing involves digitally smoothing the updated data array using a least squares polynomial regression with a five point window [20]. This non-recursive filter, originally developed by Savitzky and Golay [21], was found to be more suitable than a moving average design, which tended to degrade the resolution of smaller signals. Like the filtering routine this algorithm involves the recalculation of the central value within a moving window. The formulae and coefficients used in the algorithm are given below:

$$R_i' = \tfrac{1}{35}(-3R_{i-2} + 12R_{i-1} + 17R_i + 12R_{i+1} - 3R_{i+2})$$

where $R_i$ is the central element of the moving window and $R_i'$ is the updated element.

The effectiveness of the data processing algorithms is illustrated in figure 7 which shows the detector response for an FI peak which has had air bubbles deliberately introduced into the carrier stream. Figure 7 is a screen dump taken from a program written in-house that displays a real time graphical representation of the detector output via a serial communications link. The lower window in figure 7 also shows the peak heights of the previous injections in the analytical cycle.

After filtering and smoothing the array the peak maximum is identified and the peak height calculated by subtracting the baseline (calculated as the average of the first 10 elements of the array) from the peak maximum. After each series of injections the mean and RSD for the replicates are calculated.

### *Data validation and self diagnostics*

The validation of each pair of injections is controlled on-line by a series of simple statistical and logical tests using the system configuration variables. The routines compare the precision of the replicate injections with a minimum acceptable precision and the calculated mean peak heights with a maximum and minimum expected signal. The score given to each test is unique to that test and reflects its relative importance. Thus, poor precision is considered to be more important than either a higher or lower than expected signal. The total for all tests is assigned to an error code variable which is used to selectively trigger repeat injections by setting the re-inject flag at the appropriate value.

The time (hour and minutes), date, means of the sample and standard duplicates, calculated concentration, overall RSD and the combined error code variable are stored in the battery-backed memory at the end of each analysis.

When the monitor is idle and awaiting the next analysis, the current time, time of the next analysis and the concentration of the last measured sample are displayed on the LCD screen.

### *Detector and manifold calibration*

The performance of the solid state detector was determined by connecting a UV/visible spectrophotometer in series and running a set of absorbance standard solutions through the combined system as described above. The responses for the spectrophotometer and the solid state detector (table 1) are illustrated in figure 8. Figure 8 shows that the solid state detector (SSD) has a good correlation with the commercial spectrophotometer with a sensitivity described by the equation:

$$\text{SSD Response (counts)} = 1463 * \text{Absorbance} + 9$$

$$R^2 = 0{\cdot}9996.$$

where absorbance is the reading from the commercial spectrophotometer.

The manifold was calibrated with a series of nitrate standards covering the range 0 to 12 mg $l^{-1}$ $NO_3$-N using the optimized conditions reported previously [6]. The concentrations and corresponding SSD response are given in table 2. The calibration is linear over this range and

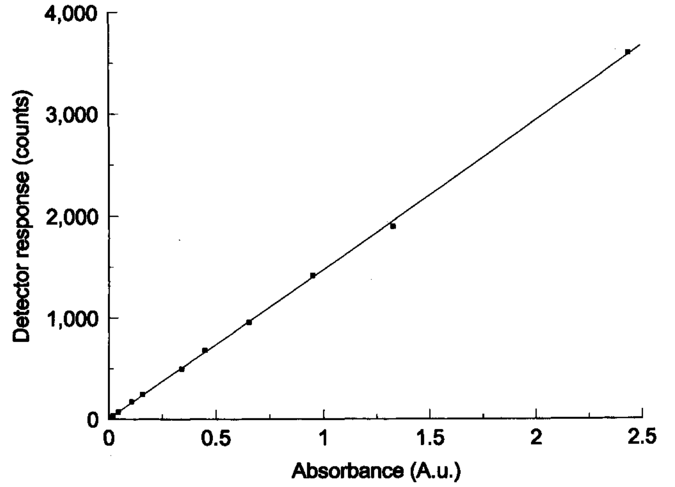

*Figure 8. Solid state detector calibration.*

*Table 2. Calibration data for the nitrate manifold.*

| | | | | | | |
|---|---|---|---|---|---|---|
| $NO_3$-N (mg $l^{-1}$) | 0 | 1 | 2 | 5 | 10 | 12 |
| Peak height (counts) | 0 | 190 | 390 | 834 | 1680 | 2040 |
| RSD (N = 6) | 2·3 | 1·1 | 0·9 | 0·9 | 1·2 | 1·0 |

*Table 3. Power consumption of the individual components of the FI monitor.*

| Component | Power consumption (mA) |
|---|---|
| Peristaltic pump (5 V) | 200 |
| Injection valve (12 V) | 400 |
| Switching valve (12 V) | 300 |
| Computer (12 V) | 40 (active) |
| | 20 (idle) |

is described by the equation:

$$\text{SSD response (counts)} = 167 * [NO_3\text{-N}(\text{mg l}^{-1})] + 19$$

$$R^2 = 0{\cdot}9993.$$

*Power consumption*

During normal operation the computer board consumes about 40 mA, which is reduced to about 20 mA during periods of monitor inactivity by placing the processor into an interrupt driven idle mode. The basic components of the FI system have the largest power consumption while the monitor is active. These data are summarized in table 3.

*Monitor trials*

The stability of the monitor and the chemistry were investigated by ratioing duplicate injections of an 8 mg $l^{-1}$ sample and a 5 mg $l^{-1}$ standard ($NO_3$-N) every 50 minutes (figure 9). The statistical data from this trial are summarized in table 4. The overall performance characteristics and reagent consumption over this seven-day trial are given in table 5.

*Table 4. Monitor performance statistics for the analysis of an 8 mg $l^{-1}$ sample and a 5 mg $l^{-1}$ standard over the seven-day laboratory trial.*

| | |
|---|---|
| Number of readings | 200 |
| Mean | 7·63[a] |
| Relative standard deviation | 0·06[a] |
| Minimum value | 7·28[a] |
| Maximum value | 7·98[a] |

[a] Units are mg $l^{-1}$ $NO_3$-N.

*Table 5. Summary of the monitor performance and reagent consumption over the seven-day laboratory trial.*

| | |
|---|---|
| Linear range | 0 to 12 mg $l^{-1}$ ($NO_3$-N) |
| Limit of detection (3σ) | 0·05 mg $l^{-1}$ ($NO_3$-N) |
| Accuracy | <5% |
| Precision | <2% |
| Carrier | 2 l |
| Colour reagent | 2 l |
| Standard | 1 l |
| Response time | 20 mins |
| Battery lifetime | >1 week |
| Data storage capacity | >3 weeks |
| Weight | 20 kg |

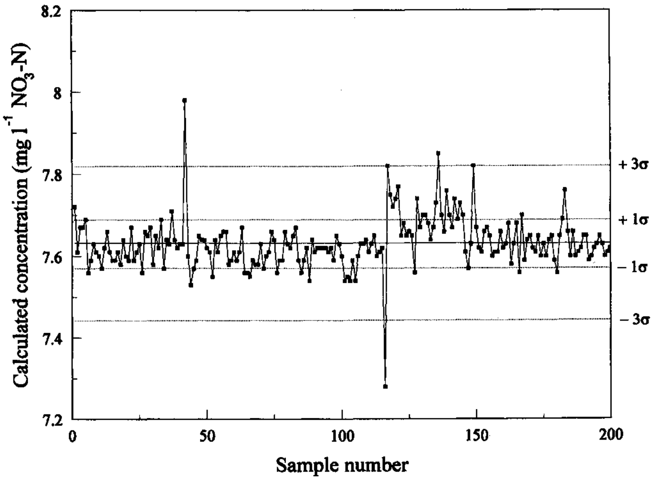

*Figure 9. Repeatability of nitrate determination over a seven-day period.*

Nitrate levels in Plymouth tapwater were measured over the period from 17 April 1993 to 21 April 1993. An unfiltered sample was fed into the system via a constant head device (200 ml capacity) and analysed every 50 minutes. No significant fluctuations above the underlying variability were observed over this period (see figure 10).

### Conclusions

The feasibility of using an FI approach for measuring water quality parameters *in situ* has been demonstrated. The FI monitor and solid state detector described perform well and are capable of measuring nitrate levels over the

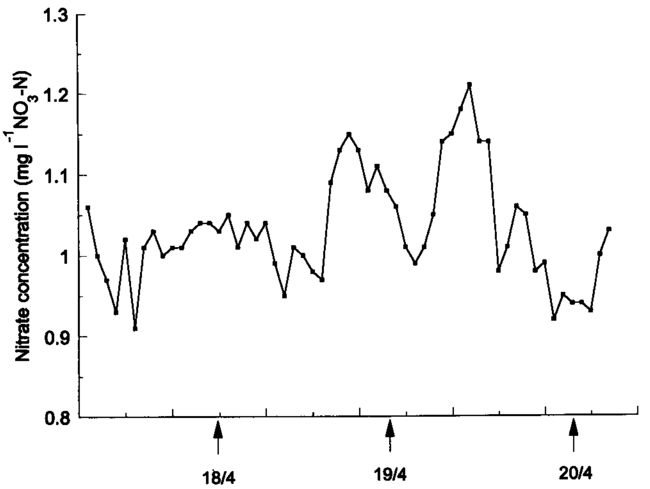

*Figure 10. Short-term fluctuation in nitrate levels in tap water from 12 p.m. on 17 April 1993 to 4 a.m. on 21 April 1993.*

range commonly encountered in freshwaters. The power supply and reagent consumption allow the monitor to operate unattended for one week. Over this period the monitor provided accurate and precise results at 50 minute intervals. The flexibility of the hardware and software design in combination with the versatility of the FI technique enable the monitor to operate in a variety of monitoring situations such as nutrient budget studies, supply intake protection and industrial process control.

### Acknowledgements

This work was funded by grant number GST/02/587 from the AFRC/NERC via a Joint Initiative on Pollutant Transport in Soils and Rocks.

### References

1. Hunt, D. T. E. and Wilson, A. L., *The Chemical Analysis of Water: General Principles and Techniques* (Royal Society of Chemistry, London, 1986).
2. See for example Gardiner, J. and Mance, G., U.K. Water Quality Standards Arising from EEC Directives, *WRc Technical Report TR204* (Medmenham, UK, 1984).
3. MacLaurin, P., Worsfold, P. J., Townshend, A., Barnett, N. W. and Crane, M., *Analyst*, **116** (1991), 701.
4. Luque de Castro, M. D. and Valcarcel, M., *International Journal of Environmental Analytical Chemistry*, **38** (1990), 171.
5. Trojanowicz, M., Benson, R. L. and Worsfold, P. J., *Trends in Analytical Chemistry*, **10**, 1 (1991), 11.
6. Clinch, J. R., Worsfold, P. J. and Casey, H., *Analytica Chimica Acta*, **200** (1987), 523.
7. Worsfold, P. J., Clinch, J. R. and Casey, H., *Analytica Chimica Acta*, **197** (1987), 43.
8. Clinch, J. R., Worsfold, P. J. and Sweeting, F., *Analytica Chimica Acta*, **214** (1988), 401.
9. Benson, R. L., Worsfold, P. J. and Sweeting, F., *Analytica Chimica Acta*, **238** (1990), 177.
10. Dasgupta, P. K., Bellamy, H. S., Liu, H., Lopez, J. L., Loree, J. L., Morris, K., Petersen, K., Kalam, A. M., *Talanta*, **40**, 1 (1993), 53.
11. Grodzik, R., *Everyday with Practical Electronics* (April 1993), 280.
12. Williams, R., *Analytical Chemistry*, **61**, 6 (1989), 433.
13. Nader, P. A. and Williams, R. R., *Analytica Chimica Acta*, **248** (1991), 285.
14. Oxidised Nitrogen in Waters, *Methods for the Examination of Waters and Associated Materials Series* (HMSO, 1981).
15. Reelsen, H., *Elektor Electronics* (May 1991), 17.
16. Trojanowicz, M., Worsfold, P. J. and Clinch, J. R., *Trends in Analytical Chemistry*, **7**, 8 (1989), 301.
17. Davies, E. R., *IEEE Proceedings E*, **139**, 2 (1992) 111.
18. Gabbouy, M., Coyle, E. J. and Gallagher, N. C., *Circuits Systems Signal Process*, **11**, 1 (1992), 7.
19. Moore, A. W. and Jorgenson, J. W., *Analytical Chemistry*, **65** (1993), 188.
20. Clark, G. D., Christian, G. D., Ruzicka, J., Anderson, G. F. and van Zee, J. A., *Analytical Instrumentation*, **18** (1989), 1.
21. Savitzky, A. and Golay, M. J. E., *Analytical Chemistry*, **36**, 8 (1964), 1627.

